# The impact of timing on outcomes in appendicectomy: a systematic review and network meta-analysis

**DOI:** 10.1186/s13017-024-00549-4

**Published:** 2024-06-14

**Authors:** Gavin G. Calpin, Sandra Hembrecht, Katie Giblin, Cian Hehir, Gavin P. Dowling, Arnold D.K. Hill

**Affiliations:** 1 Department of Surgery Beaumont Hospital, Dublin, Ireland; 2 Royal College of Surgeons in Ireland, 123 St Stephens Green Dublin 2, Dublin, Ireland

**Keywords:** Appendicitis, Appendicectomy, Timing, Outcomes, Systematic review

## Abstract

**Introduction:**

Appendicectomy remains the standard treatment for appendicitis. There is a lack of clarity on the timeframe in which surgery should be performed to avoid unfavourable outcomes.

**Aim:**

To perform a systematic review and network meta-analysis to evaluate the impact the (1)time-of-day surgery is performed (2), time elapsed from symptom onset to hospital presentation (patient time) (3), time elapsed from hospital presentation to surgery (hospital time), and (4)time elapsed from symptom onset to surgery (total time) have on appendicectomy outcomes.

**Methods:**

A systematic review was performed as per PRISMA-NMA guidelines. The time-of-day which surgery was done was divided into day, evening and night. The other groups were divided into < 24 h, 24–48 h and > 48 h. The rate of complicated appendicitis, operative time, perforation, post-operative complications, surgical site infection (SSI), length of stay (LOS), readmission and mortality rates were analysed.

**Results:**

Sixteen studies were included with a total of 232,678 patients. The time of day at which surgery was performed had no impact on outcomes. The incidence of complicated appendicitis, post-operative complications and LOS were significantly better when the hospital time and total time were < 24 h. Readmission and mortality rates were significantly better when the hospital time was < 48 h. SSI, operative time, and the rate of perforation were comparable in all groups.

**Conclusion:**

Appendicectomy within 24 h of hospital admission is associated with improved outcomes compared to patients having surgery 24–48 and > 48 h after admission. The time-of-day which surgery is performed does not impact outcomes.

**Supplementary Information:**

The online version contains supplementary material available at 10.1186/s13017-024-00549-4.

## Introduction

Appendicitis remains a prevalent surgical emergency worldwide, necessitating prompt intervention to mitigate the risk of complications. Appendicectomy is widely considered the gold standard treatment of acute appendicitis [1, 2]. While the urgency of surgical intervention is widely acknowledged, the optimal timing of appendicectomy remains a subject of ongoing debate within the surgical community. In recent years, evidence has suggested that the timing of appendicectomy may play a crucial role in determining patient outcomes, influencing factors such as postoperative complications, length of hospital stay, and overall recovery [3–5].

A recent randomised control trial (RCT) by Jalava et al. found that the rate of appendiceal perforation when surgery was performed within 24 h was comparable to that performed within 8 h [6]. However, there is a general consensus that delaying surgery is associated with worse outcomes and inferior outcomes have been reported when surgery is performed more than 24 h after hospital presentation [7]. Current guidelines recommend performing appendicectomy as soon as possible [8, 9] or within 24 h [10] but there are no large-scale randomised studies which have informed these recommendations. Therefore, it is unclear the exact timeframe in which surgery should be performed in acute appendicitis to obtain preferable outcomes.

Nevertheless, the landscape of appendicitis management continues to evolve, and this review intends to integrate recent evidence to inform contemporary clinical practice. By synthesising data from a diverse range of studies, we seek to elucidate the relationships between timing of appendicectomy and clinical outcomes. The nuanced interplay between timing and outcomes is a critical aspect that warrants careful examination. By employing a network meta-analysis approach, we aim to compare different timeframes for appendicectomy and discern their relative efficacy. Furthermore, we wish to determine whether appendicectomy in the evening and night time has an impact on outcomes when compared with surgery during the daytime.

In conclusion, we aimed to perform a systematic review and network meta-analysis (NMA) to determine the impact which the timing of appendicectomy has on clinical outcomes. By consolidating the available evidence, we strived to contribute to the ongoing discourse surrounding optimal appendicitis management, providing clinicians with evidence-based insights to guide decision-making and enhance patient care.

## Methods

This systematic review was performed in accordance to the Preferred Reporting Items for Systematic Reviews and Meta-Analyses-Network Meta-Analyses (PRISMA-NMA) [11] checklist and the Cochrane Handbook for Systematic Reviews and Intervention [12]. All authors contributed to formulating the study protocol and it was then registered with the International Prospective Register of Systematic Reviews (PROSPERO)(CRD42024502346). Local institutional ethical approval was not required. All authors declare no conflicts of interest. This research received no external funding.

### PICO

Using the PICO framework [13], the aspects the authors wished to address were:

Population – Any patient with appendicitis who underwent appendicectomy.

Intervention – Any patient who underwent appendicectomy during the daytime, presented to hospital within 24 h of symptom onset (patient time), had surgery within 24 h of hospital presentation (hospital time) or had surgery within 24 h of symptom onset (total time).

Comparison – Any patient who underwent appendicectomy in the evening or night time, had a patient time of 24–48 h or > 48 h, had a hospital time of 24–48 h or > 48 h, or had a total time of 24–48 h or > 48 h.

Outcomes – The rate of complicated appendicitis, operative time, perforation, post-operative complications, surgical site infection (SSI), length of stay (LOS), readmission and mortality rates within each group.

### Search strategy

An electronic search was performed of the *PubMed Medline, Scopus, and Embase* databases for relevant studies. This search was performed by two independent reviewers (GGC & SH), using a predetermined search strategy that was designed by senior author (ADKH). This search included the search terms: (appendectomy), (appendicectomy), (timing), and (outcomes) with ‘AND’ and ‘OR’ as a Boolean operators. Included studies were limited to the English language. The search was not restricted by year of publication. All duplicate studies were manually removed, before titles were screened, and studies considered appropriate had their abstracts and/or full text reviewed. Retrieved studies were reviewed to ensure inclusion criteria were met for one outcome at a minimum. In cases of discrepancies of opinion a third author was asked to arbitrate (KG). The reference lists of included studies were also screened for additional relevant studies. The final search was performed on the 26th January 2024.

### Inclusion criteria

Studies included in this network meta-analysis fulfilled the following criteria:

1. All patients had appendicitis and underwent appendicectomy.
2. The timing of surgery in at least two groups were compared.
3. At least one outcome of interest was reported.
4. Full text available for review.

### Exclusion criteria

Studies excluded from this network meta-analysis fulfilled the following criteria:

1. The groups compared had different timepoints to those analysed in this study.
2. Reported on different outcomes.
3. Only abstract available for review.
4. Published abstracts from conference proceedings, review articles, case reports, and editorial articles.

### Data extraction

The following data was extracted and collated from retrieved studies meeting inclusion criteria: (1) First author name, (2) year of publication, (3) country of origin, (4) study type, (5) journal, (6) number of patients, (7) outcomes of interest.

### Statistical analysis

Descriptive statistics were used to outline characteristics of included trial data. Rates of complicated appendicitis, perforation, post-operative complications, SSI, readmission and mortality were expressed as binary outcomes, reported as odds ratios (ORs) and effect sizes were described with a 95% confidence interval (CI). Operative time and LOS were expressed as continuous outcomes, reported as mean differences (MDs) with standard deviations (SD) and effect sizes were described with a 95% credible interval (CrI). The principal comparator was ‘daytime’ in the time of day analyses and < 24 h in the patient time, hospital time and total time analyses.

Bayesian NMAs were conducted using netameta and Shiny packages for R [14]. Results were considered statistically significant at the *P* < 0.050 level if the 95% CI did not include the value of one and if the 95% CrI did not include the value of zero. The Mantel-Haenszel method was employed to perform meta-analysis, where necessary, using Review Manager [15], Version 5.4 (Nordic Cochrane Centre, Copenhagen, Denmark). Risk of bias and methodology quality assessment was performed in accordance with the Cochrane risk of bias assessment guidelines using the ROBINS-I tool [16]. ‘GRADEpro’ was used to assess the quality of evidence.

## Results

### Literature search

**Fig. 1:**
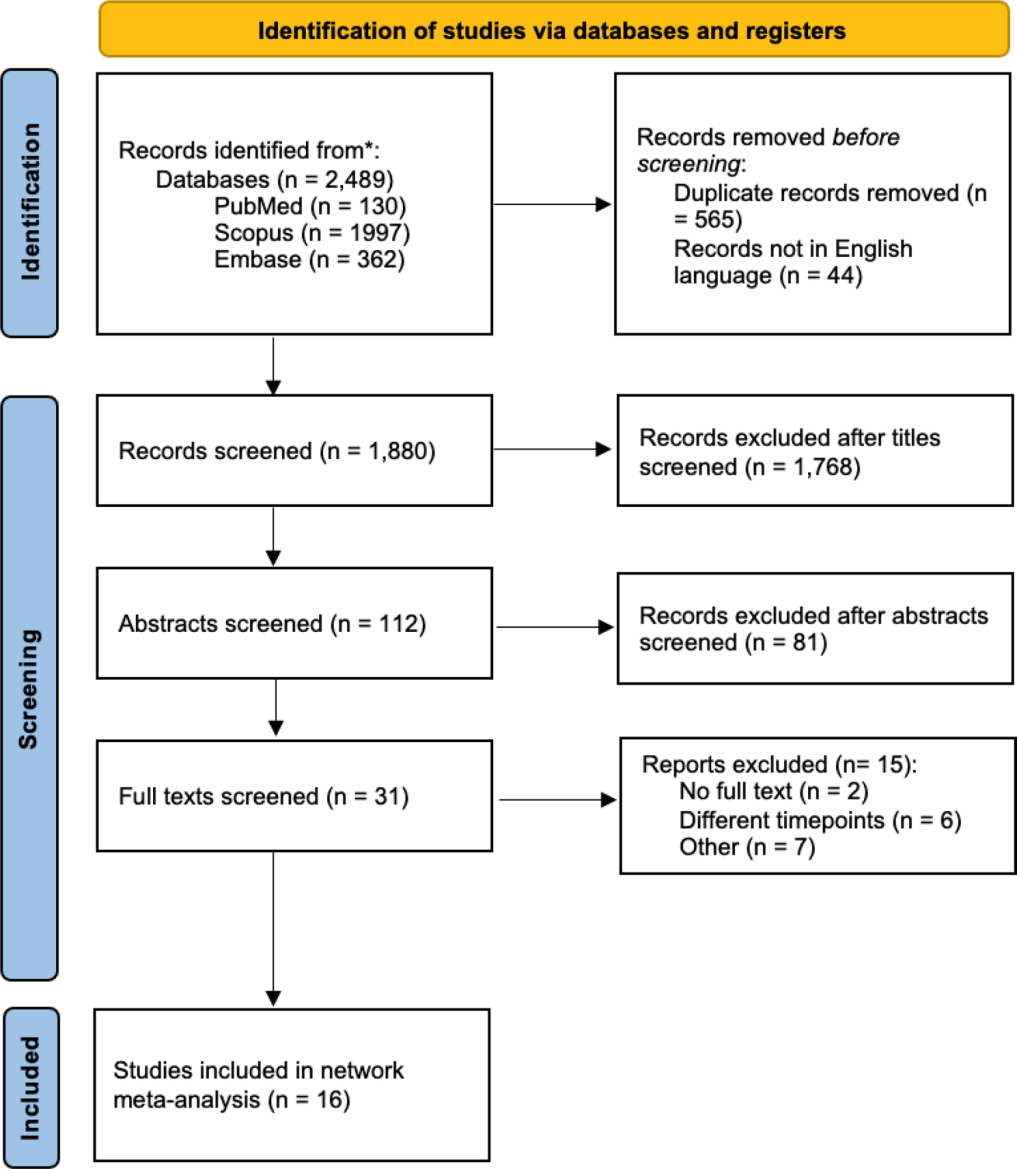
PRISMA flow diagram of study identification

Overall, 2,489 studies were identified in the database search. After duplicates and non-English texts were removed, there were 1,880 articles left. Following screening of titles and abstracts, 31 full texts were left to be reviewed. Of these, 15 were excluded leaving 16 studies for inclusion in the network meta-analysis. The search strategy and study identification are summarised in the PRISMA flow diagram (Fig. 1).

### Study and patient characteristics

There were 16 studies included in the NMA [7, 17–31]. Four studies compared outcomes based on the time-of-day of when surgery was performed [17, 23, 27, 31]. Three studies compared outcomes based on patient time [18, 19, 24]. Eleven studies compared outcomes based on hospital time [20–29, 31]. Four studies compared outcomes based on total time [7, 18, 19, 30]. Thirteen studies were retrospective and three were prospective [7, 18, 30]. Publication dates ranged from 2012 to 2023 and surgery dates ranged from 2003 to 2021. Study data from the included studies is outlined in Table 1. Risk of bias assessments are outlined in Supplementary Materials 1 and 2 and GRADE assessments are outlined in Supplementary Materials 7 and 8.

**Table 1:** Study characteristics

Author & Year	Country	Journal	Study type	Enrolment period	Number of patients	Time analysed
San Basilio 2023	Spain	Paediatr Surg	Retrospective	2017–2021	1,643	Time-of-day
Mandeville 2015	USA	Pediatr Emerg Care	Prospective	2009–2010	230	Patient time Total time
Kim 2015	Korea	Ann Surg Treat Res	Retrospective	2013	192	Patient time Total time
Giraudo 2013	Italy	Surg Today	Retrospective	2003–2009	723	Hospital time
Chen 2015	Taiwan	Journal of the Chinese Medical Association	Retrospective	2010–2012	236	Hospital time
Teixeira 2012	USA	Ann Surg Treat Res	Retrospective	2003–2011	4,108	Hospital time
Bonadio 2015	USA	J Emerg Med	Retrospective	2010–2014	248	Hospital time
Patel 2018	Canada	J Trauma Acute Care Surg	Retrospective	2009–2015	25,874	Time-of-day Hospital time
Alore 2018	USA	J Surg Res	Retrospective	2012–2015	112,122	Hospital time
Andert 2017	Germany	Langenbecks	Retrospective	2003–2014	2,136	Hospital time
Fair 2015	USA	AM J Surg	Retrospective	2007–2012	69,926	Hospital time
Canal 2020	Switzerland	Int J Surg	Retrospective	2010–2017	9,224	Time-of-day Hospital time
Saar 2016	Estonia	World J Surg	Prospective	2013–2014	266	Total time
Jiang 2021	China	Asian J Endosc Surg	Prospective	2017–2019	255	Total time
Almström 2017	Sweden	Ann Surg	Retrospective	2006–2013	2,756	Time-of-day Hospital time
Ashkenazi 2022	Israel	Eur J Trauma Emerg Surg	Retrospective	2006–2016	2,749	Patient time Hospital time

In total, there was data from 232,678 patients included from the 16 studies. Of the patients included, 97.9% were adult and 2.1% were paediatric. Laparoscopic appendicectomy was performed in 85.5% of cases while open surgery was adopted in 14.5% of cases. Complicated appendicitis was found in 28.5% (14,442/50,640) of cases (Table 2).

**Table 2:** Number of patients with complicated and uncomplicated appendicitis

Author & Year	Number of patients	Uncomplicated (%)	Complicated (%)
San Basilio 2023	1,643	945 (57.5%)	698 (42.5%)
Mandeville 2015	230	166 (72.2%)	64 (27.8%)
Kim 2015	192	94 (49.0%)	98 (51.0%)
Giraudo 2013	723	586 (81.1%)	137 (18.9%)
Chen 2015	236	206 (87.3%)	30 (12.7%)
Teixeira 2012	4,108	3,166 (77.1%)	942 (22.9%)
Bonadio 2015	248	248 (100.0%)*	-
Patel 2018	25,874	16,508 (63.8%)	9,366 (36.2%)
Alore 2018	112,122	-	-
Andert 2017	2,136	1,697 (79.4%)	439 (20.6%)
Fair 2015	69,926	-	-
Canal 2020	9,224	8,085 (87.7%)	1,139 (12.3%)
Saar 2016	266	217 (81.6%)	49 (18.4%)
Jiang 2021	255	206 (80.8%)	49 (19.2%)
Almström 2017	2,756	2,095 (76.0%)	661 (24.0%)
Ashkenazi 2022	2,749	1,979 (72.0%)	770 (28.0%)

*Only patients with uncomplicated appendicitis on radiological imaging (US/CT) were included, but 54 (21.8%) developed a perforation

### Outcomes

#### Time-of-day which surgery was performed (day, evening, night)

There were 39,497 patients included in this analysis. Surgery was performed during the daytime in 36.5% (14,419) of cases, in the evening time in 49.1% (19,410) of cases, and overnight in 14.4% (5,668) of cases.

**Fig. 2:**
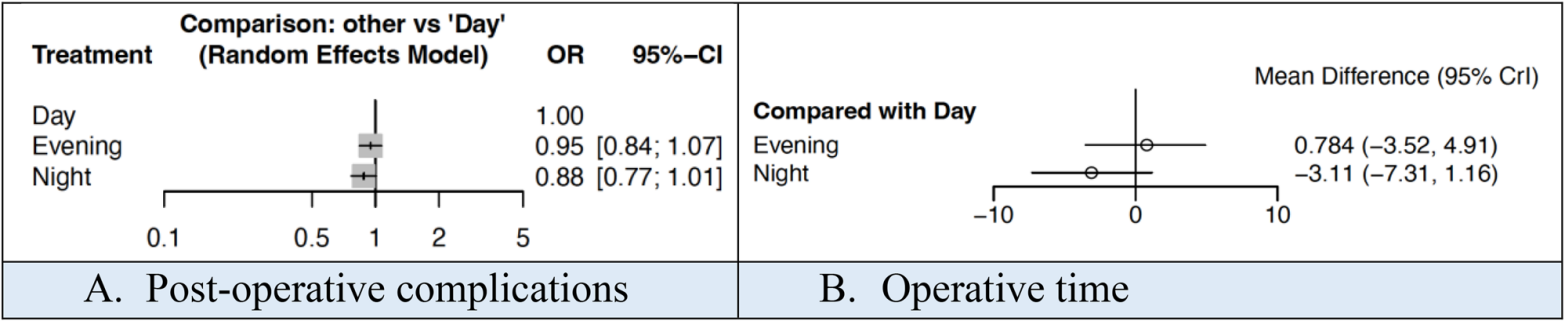
Forest plots comparing outcomes based on the time-of-day surgery was performed

The incidence of post-operative complications was 32.9%, 40.7%, 34.2% when surgery was performed during the daytime, evening time and night time respectively. The mean operative time was 64.0, 62.0 and 57.4 min respectively. There was no statistically significant difference in either of these outcomes (Fig. 2). NMA league ranking charts for outcomes based on time-of-day surgery was performed are outlined in Supplementary Material 3.

#### Patient time (symptom onset to hospital presentation)

There were 2,982 patients included in this analysis. Patients presented to hospital within 24 h of symptom onset in 51.4% (1,534) of cases, 24–48 h after symptom onset in 20.5% (612) of cases and more than 48 h after symptom onset in 28.0% (836) of cases.

**Fig. 3:**
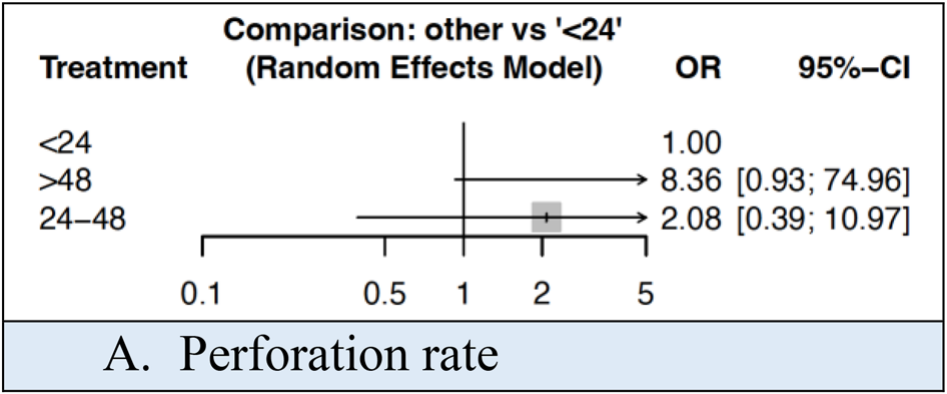
Forest plot comparing outcomes based on patient time

The perforation rate was 30.4%, 40.5% and 48.4% in those with a patient time of < 24 h, 24–48 h and > 48 h respectively. This was not statistically significant at network meta-analysis (Fig. 3). NMA league ranking charts for outcomes based on patient time are outlined in Supplementary Material 4.

#### Hospital time (hospital presentation to surgery)

There were 229,224 patients included in this analysis. Surgery was performed within 24 h of hospital presentation in 79.9% (183,133) of cases, 24–48 h after hospital presentation in 19.2% (44,098) of cases, and more than 48 h after hospital presentation in 0.9% (1,993) of cases.

The incidence of perforation was 25.8%, 22.3% and 36.0% in patients with a hospital time < 24 h, 24–48 h and > 48 h respectively. The mean operative time was 60.6 min and 69.4 min in patients with a hospital time < 24 h and 24–48 h respectively. Neither of these were statistically significant.

**Fig. 4:**
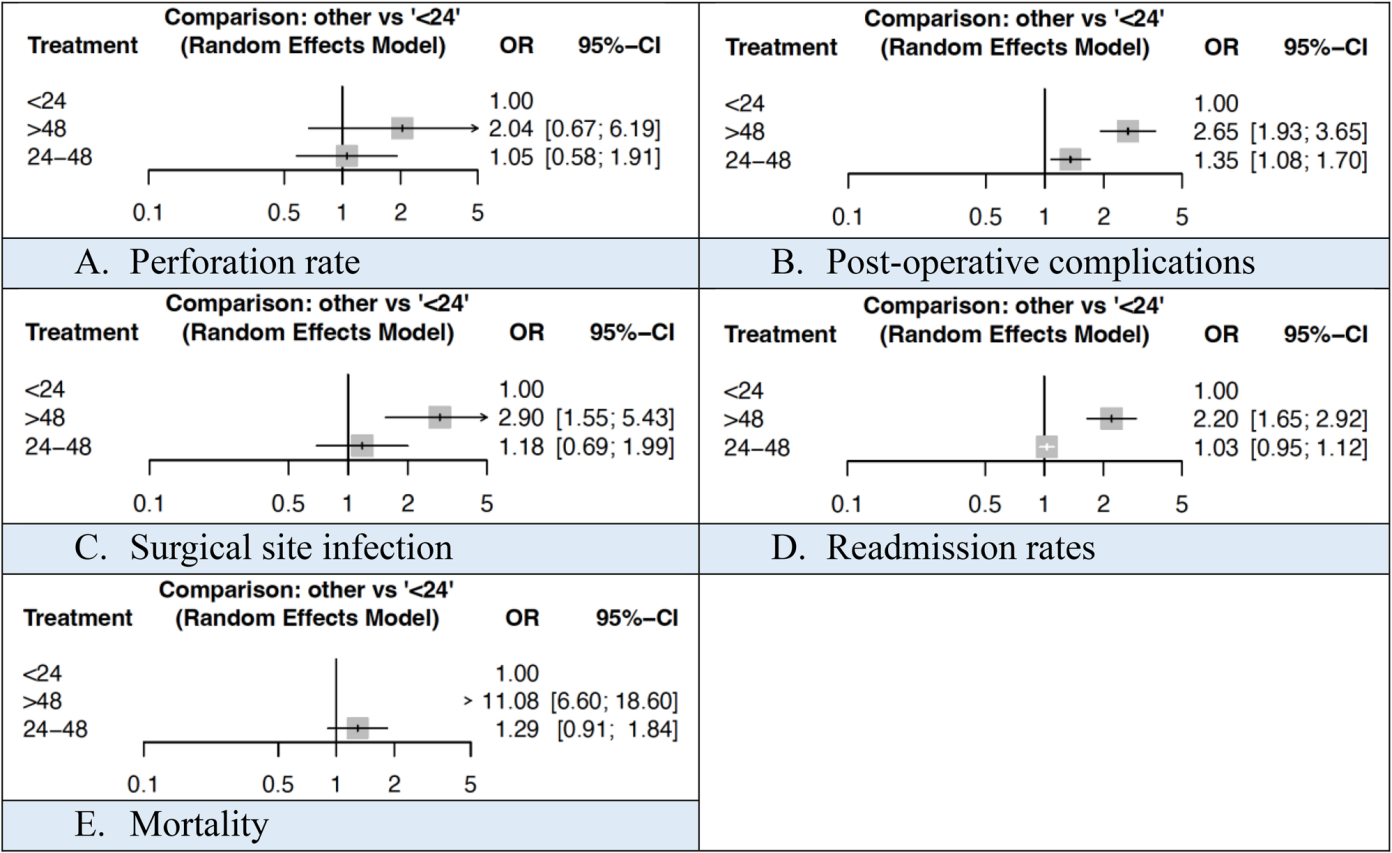
Forest plots comparing outcomes based on hospital time

**Fig. 5:**
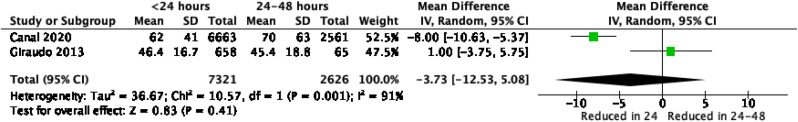
Operative time based on hospital time

**Fig. 6:**
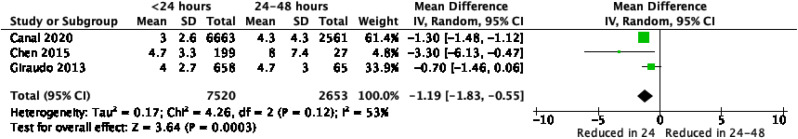
Length of stay based on hospital time

Post-operative complications occurred in 11.7%, 10.5% and 15.3% in patients with a hospital time < 24 h, 24–48 h and > 48 h respectively. SSI occurred in 0.9%, 1.0% and 1.8% respectively. Readmission rates were 2.9%, 3.0% and 6.1% respectively. Mortality rates were < 0.1%, 0.1% and 0.9% respectively. The mean LOS was 3.1 days and 4.3 days in patients with a hospital time < 24 h and 24–48 h respectively. All these analyses were statistically significant (Figs. 4, 5 and 6). NMA league ranking charts for outcomes based on hospital time are outlined in Supplementary Material 5.

#### Total time (symptom onset to surgery)

There were 933 patients included in this analysis. Surgery was performed within 24 h of symptom onset in 33.3% (311) of cases, 24–48 h after symptom onset in 38.8% (362) of cases, and more than 48 h after symptom onset in 27.9% (1,993) of cases.

**Fig. 7:**
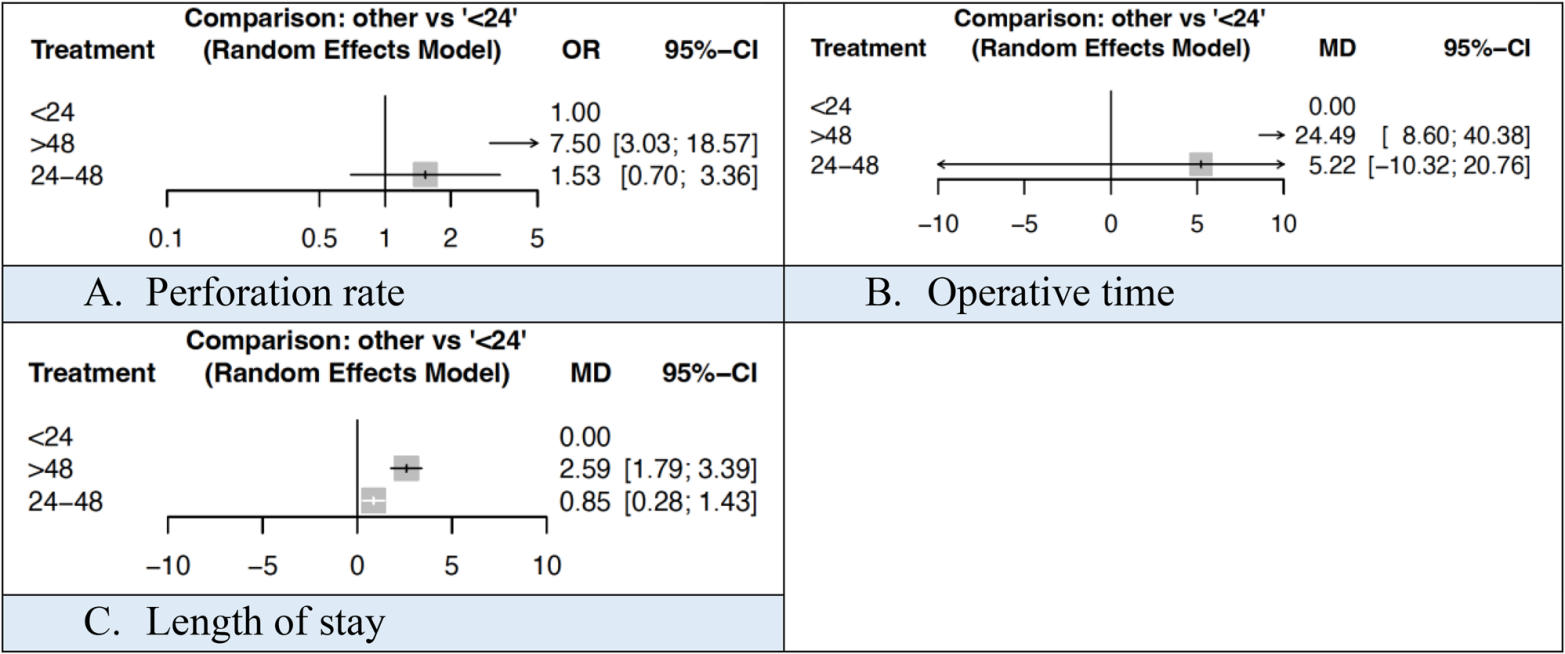
Forest plots comparing outcomes based on total time

The rate of perforation was 25.3%, 29.9% and 40.6% in patients with a total time < 24 h, 24–48 h and > 48 h respectively. The mean operative time was 42.1, 53.1 and 79.2 min respectively and the mean LOS was 2.1, 3.4 and 5.4 days respectively. These were statistically significant at network meta-analysis (Fig. 7). NMA league ranking charts for outcomes based on total time are outlined in Supplementary Material 6.

## Discussion

Appendicectomy remains the gold standard of treatment for acute appendicitis, with the laparoscopic approach being the increasingly performed approach over time with decreasing rates of conversion to open surgery [32]. This systematic review encompassed 232,678 patients undergoing appendicectomy over 16 studies, assessing the impact of timing on clinical outcomes, and so, conclusions can be made regarding the four aims outlined at the beginning of the paper. The results garnered can guide significantly the way appendicitis should be managed in the acute hospital setting.

### Out of hours surgery

Previously published, retrospective multi-centred cohort studies and meta-analyses have demonstrated that patients undergoing undifferentiated surgical procedures between the hours of 17:00 and 07:00 have higher rates of morbidity and mortality [33, 34]. This has been previously felt to be related to surgeon fatigue as well as other patient related factors. Evidence from these type of papers has been used to justify not proceeding with appendicectomy late at night and postponing appendicectomy until the following morning with the justification of decreased patient safety. This study indicates that patients undergoing appendicectomy have similar rates of post operative complications regardless of time of day the operation is carried out with no statistically significant difference in mean operative time. Furthermore, this data has shown that delays from time of hospital presentation to surgery negatively impacted rates of post operative complications, hospital readmission rates, mortality rates and length of stay. Surgery for appendicitis should be performed promptly upon confirmation of diagnosis. In keeping with the findings of Jalava et al. [6] and van Dijk et al. [35], surgery for presumed uncomplicated appendicitis can wait until the morning after if the hospital time is < 24 h. However, it appears safe to proceed with surgery ‘out-of-hours’ if it is felt that the patient may benefit clinically so to ensure best potential outcomes for patients.

### Out of hours access to diagnostic imaging

Results from this systematic review indicate that delays in access to definitive surgery from time of hospital admission result in statistically significant increases in post-operative complications and surgical site infection. Another major source of delay following admission is access to appropriate diagnostic testing. In an era of defensive medicine, surgeons are becoming increasingly reliant on confirmatory diagnostic imaging tests prior to proceeding with surgical intervention, including for appendicitis. Out of hours access to user dependent modalities such as ultrasound can be severely limited and computed tomography (CT) is increasingly becoming the imaging modality of choice [36]. Access to CT scanning is not guaranteed and as a finite resource, particularly out of hours, patients are triaged based on urgency of need. Predictive tests such as Alvarado or AIR scores are insufficiently sensitive to be used in isolation and the World Society of Emergency Surgery recommend they are used in conjunction with ultrasonography or cross sectional imaging to appropriately guide management [37]. Increasing availability of out of hours diagnostic imaging allowing for prompt surgical intervention in appropriate patients will improve patient outcomes.

### Healthcare resourcing

We have also showed that delays in undergoing surgery from time of symptom onset result in statistically significant increases in rates of perforation, operative time and hospital length of stay. Patient’s access to primary care review and referral to an appropriate surgical centre can impact this delay. Similarly, ongoing reporting of hospital overcrowding on media outlets act as a deterrent for patients to attend emergency departments on certain days of the week and time of the day. Increasing access to healthcare in the primary and urgent care setting will improve these outcomes.

Overall, these results have showed that striving for prompt diagnosis and surgical management of acute appendicitis results in improved outcomes for patients. Developing pathways which streamline the patient journey from primary care to operating theatre with ringfenced access to appropriate diagnostic testing and short stay surgical wards would improve outcomes and provide more cost effective management of one of the most common general surgical emergency presentations.

### Complicated and uncomplicated appendicitis

Perhaps, one of the most important clinical considerations in these patients is the presence or absence of complicated appendicitis. There is evidence to suggest that most uncomplicated cases settle spontaneously within 24 h [38, 39] whereas complicated cases need surgical intervention in the majority of cases. On the other hand, the COMMA trial showed that 25.3% of patients treated conservatively for uncomplicated appendicitis experienced a recurrence within 1 year and a higher quality of life was found in patients who underwent surgery [2]. Therefore, the authors feel that surgery should remain the mainstay of treatment for appendicitis even if it is uncomplicated.

There is difficulty interpreting the results of this analysis as complicated appendicitis was found in 28.5% of cases. The impact which this has on the results of the analysis is unclear. Nine studies reported on outcomes according to the presence or absence of complicated appendicitis [19, 21, 22, 24–27, 30, 31]. Unsurprisingly, outcomes were worse in cases of complicated appendicitis. However, we were unable to perform subgroup analyses of uncomplicated and complicated appendicitis separately as this data was not reported in any of the included studies. Therefore, there is a gap in the conclusions which can be drawn from this study. It is possible that patients with uncomplicated appendicitis can obtain optimal outcomes even if surgery is performed 24–48 h after admission but unfortunately, there is a lack of large-scale evidence to determine this. An interesting finding in Bonadio et al. was the development of a perforation in 21.8% of patients with uncomplicated appendicitis despite parenteral antibiotics [26]. Independent factors associated with perforation included longer waiting times for surgery (41% after 24 h), the presence of a fever and the presence of an appendicolith.

Considering current evidence, the authors feel that patients with complicated appendicitis should have their surgeries prioritised. Those with uncomplicated appendicitis do not require intervention as urgently but may have inferior outcomes if surgery is performed more than 24 h after hospital presentation. Surgeons should use clinical acumen to best allocate operating room resources for those with uncomplicated appendicitis. Factors which may heighten the risk of perforation in these cases include surgery > 24 h after admission, fever, and the presence of an appendicolith. In cases where surgery for uncomplicated appendicitis is likely to occur 24–48 h after hospital presentation, patients should be administered antibiotics.

### Strengths and limitations

This study is subject to a number of limitations. Firstly, many of the included studies are retrospective in design and are, therefore, inherently prone to confounding biases. Furthermore, this also means there is significant heterogeneity. Statistical heterogeneity underlying the analysis is an important consideration. Thus, the results must be interpreted with caution. influences an important inconsistence Furthermore, trials cannot be blinded, predisposing to a potential for performance bias. Patients who presented with complicated appendicitis are likely to have had their surgery prioritised and performed sooner than those with uncomplicated appendicitis and this is likely to have impacted our results. It is also worth noting that our analysis includes both paediatric and adult patients and a subgroup analysis could not be performed to determine if there is any difference in outcomes in the two cohorts.

Nevertheless, this study includes data from a large number of patients. To the knowledge of the authors, it is the first network meta-analysis of the timing of surgery in appendicitis and provides a comprehensive update on the systematic review published by van Dijk et al. [35]. Not only does this network meta-analysis highlight outcomes based on hospital time, but it also evaluates outcomes in relation to patient time, total time and the time-of-day which surgery was performed. The wide range of outcomes analysed highlights the comprehensiveness of the study and is an addition to current literature. We should aim to minimise intravenous antibiotic requirements, complications, and length of stay to improve patient outcomes, reduce hospital cost, and increase the availability of healthcare resources. We know that appendiceal perforation rates are comparable at hospital times of 8 h and 24 h [6], but the findings of our analysis suggest that, where possible, surgery should not be delayed beyond this and in this regard, acts as an addendum to the RCT by Jalava et al. [6].

## Conclusion

In conclusion, this systematic review and network meta-analysis shows that appendicectomy within 24 h of hospital presentation is associated with improved outcomes compared to surgery performed 24–48 h and > 48 h afterwards. It does not appear to be affected by the time-of-day at which surgery is performed. The results of this systematic review support the conclusions of the most recent randomised control trial. However, the heterogenous nature of included studies means we must be interpret the findings with caution.

### Electronic supplementary material

Below is the link to the electronic supplementary material.

Supplementary Material 1

Supplementary Material 2

Supplementary Material 3

## Data Availability

No datasets were generated or analysed during the current study.

## Abbreviations

RCT: Randomised control trial
NMA: Network meta-analysis
SSI: Surgical site infection
LOS: Length of stay
