# Supplementary material for: The impact of timing on outcomes in appendicectomy: a systematic review and network meta-analysis
Source: World J Emerg Surg. 2024 Jun 14;19:24. doi: 10.1186/s13017-024-00549-4 (PMC11177546; doi:10.1186/s13017-024-00549-4)
Supplement: Supplementary file 1 — Supplementary Material 1 [file 13017_2024_549_MOESM1_ESM.docx]

- An electronic search was performed of the *PubMed Medline, Scopus, and Embase* databases for relevant studies.
- This search was performed by two independent reviewers (GGC & SH), using a predetermined search strategy that was designed by senior author (ADKH). In cases of discrepancies of opinion a third author was asked to arbitrate (KG).
- This search included the search terms: (appendectomy), (appendicectomy), (timing), and (outcomes) with ‘AND’ and ‘OR’ as a Boolean operators.
- Included studies were limited to the English language.
- The search was not restricted by year of publication.
- All retrieved studies were exported and compiled into an Excel spreadsheet.
- All duplicate studies were manually removed and then non-English texts were removed.
- Titles were screened and studies considered appropriate had their abstracts reviewed.
- Studies which were considered relevant after abstract review were included for full text review.
- Retrieved studies were reviewed to ensure inclusion criteria were met for one outcome at a minimum.
- The final search was performed on the 26^th^ January 2024.
- The following data was extracted and collated from retrieved studies meeting inclusion criteria: First author name, year of publication, country of origin, study type, journal, number of patients, timing of surgery (<24 hours, 24-48 hours, >48 hours), outcomes of interest.
