# Supplementary material for: The impact of timing on outcomes in appendicectomy: a systematic review and network meta-analysis
Source: World J Emerg Surg. 2024 Jun 14;19:24. doi: 10.1186/s13017-024-00549-4 (PMC11177546; doi:10.1186/s13017-024-00549-4)
Supplement: Supplementary file 2 — Supplementary Material 2 [file 13017_2024_549_MOESM2_ESM.docx]

| **Certainty assessment** | | | | | | | **№ of patients** | | **Effect** | | **Certainty** | **Importance** |
| --- | --- | --- | --- | --- | --- | --- | --- | --- | --- | --- | --- | --- |
| **№ of studies** | **Study design** | **Risk of bias** | **Inconsistency** | **Indirectness** | **Imprecision** | **Other considerations** | **Appendicectomy <24 hours** | **Appendicectomy >24 hours** | **Relative (95% CI)** | **Absolute (95% CI)** |  |  |
| **Length of stay (days)** | | | | | | | | | | | | |
| 3 | non-randomised studies | not serious | not serious | not serious | not serious | none | 7520 | 2653 | - | MD **0.7 days lower** (1.83 lower to 0.55 lower) | ⨁⨁⨁⨁ High |  |
| **Perforation** | | | | | | | | | | | | |
| 4 | non-randomised studies | not serious | not serious | not serious | not serious | all plausible residual confounding would reduce the demonstrated effect | 1451/5619 (25.8%) | 178/798 (22.3%) | **OR 1.05** (0.58 to 1.91) | **9 more per 1,000** (from 80 fewer to 131 more) | ⨁⨁⨁⨁ High |  |
| **Operative time** | | | | | | | | | | | | |
| 2 | non-randomised studies | not serious | not serious | not serious | not serious | none | 7321 | 2626 | - | MD **3.73 minutes lower** (12.53 lower to 5.08 lower) | ⨁⨁⨁⨁ High |  |
| **Post-operative complications** | | | | | | | | | | | | |
| 6 | non-randomised studies | not serious | serious | not serious | not serious | none | 20384/174564 (11.7%) | 4525/43004 (10.5%) | **OR 1.35** (1.08 to 1.70) | **3,178 more per 100,000** (from 747 more to 6,138 more) | ⨁⨁⨁◯ Moderate |  |
| **Surgical site infection** | | | | | | | | | | | | |
| 3 | non-randomised studies | not serious | not serious | not serious | not serious | none | 1378/146364 (0.9%) | 384/38023 (1.0%) | **OR 1.18** (0.69 to 1.99) | **18 more per 10,000** (from 31 fewer to 98 more) | ⨁⨁⨁⨁ High |  |
| **Readmission rates** | | | | | | | | | | | | |
| 2 | non-randomised studies | not serious | not serious | not serious | not serious | none | 2609/88706 (2.9%) | 745/24681 (3.0%) | **OR 1.03** (0.95 to 1.12) | **9 more per 10,000** (from 15 fewer to 35 more) | ⨁⨁⨁⨁ High |  |
| **Mortality** | | | | | | | | | | | | |
| 2 | non-randomised studies | not serious | not serious | not serious | not serious | none | 121/142848 (0.1%) | 41/37692 (0.1%) | **OR 1.29** (0.91 to 1.84) | **3 more per 10,000** (from 1 fewer to 9 more) | ⨁⨁⨁⨁ High |  |

**CI:** confidence interval; **MD:** mean difference; **OR:** odds ratio

Supplementary Material 7: GRADE assessment


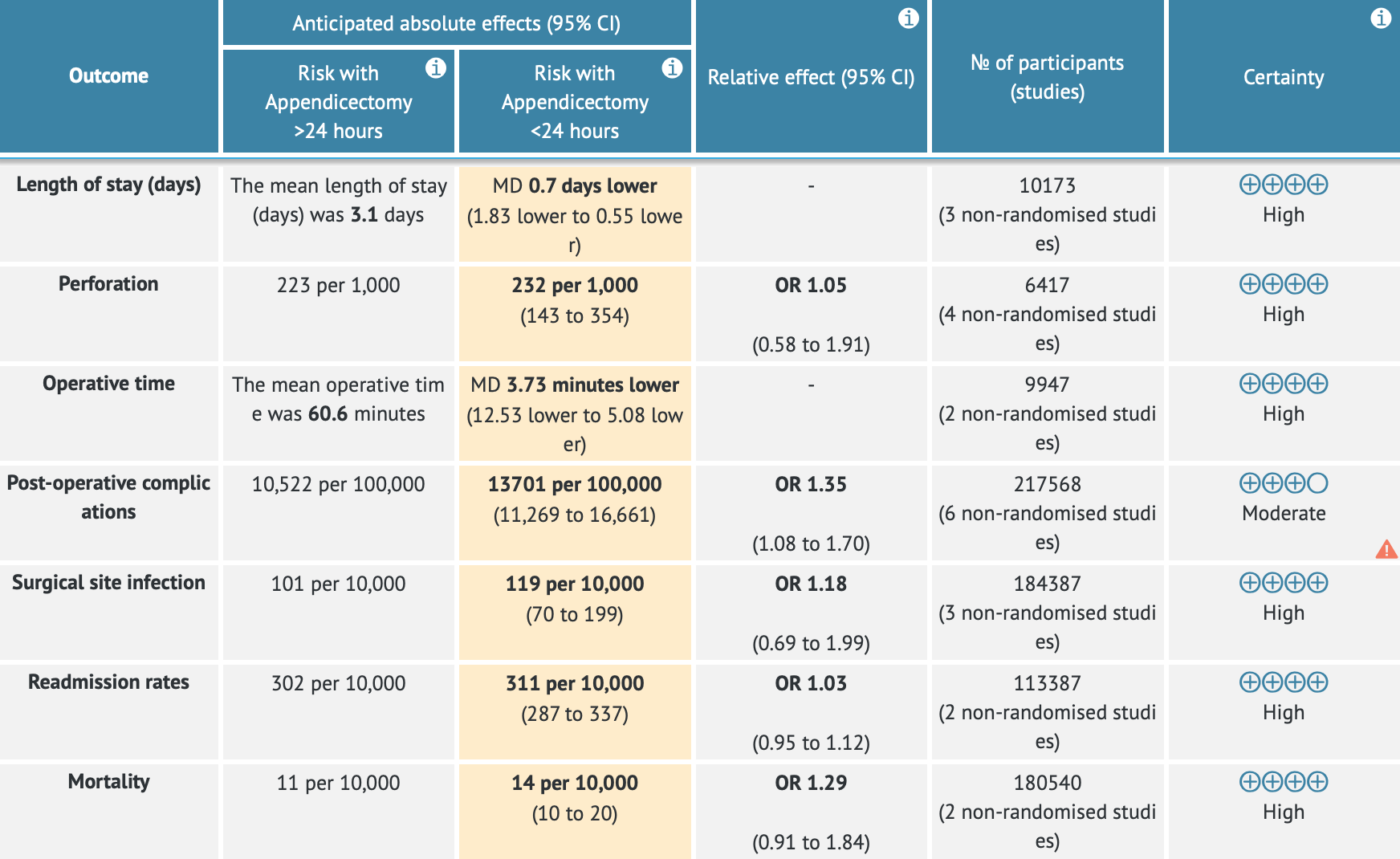


Supplementary Material 8: Summary table of GRADE assessment
