# Supplementary material for: The impact of timing on outcomes in appendicectomy: a systematic review and network meta-analysis
Source: World J Emerg Surg. 2024 Jun 14;19:24. doi: 10.1186/s13017-024-00549-4 (PMC11177546; doi:10.1186/s13017-024-00549-4)
Supplement: Supplementary file 3 — Supplementary Material 3 [file 13017_2024_549_MOESM3_ESM.docx]

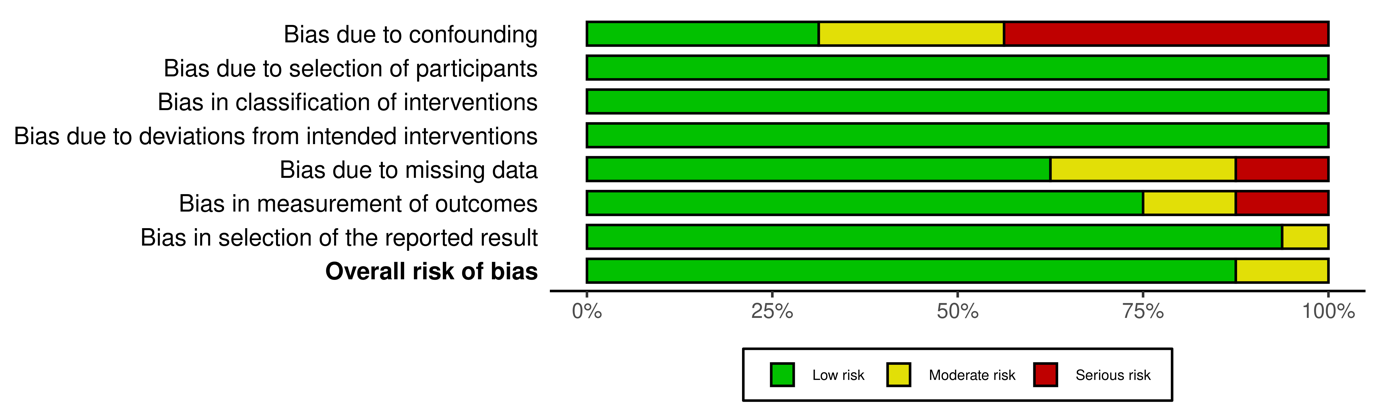


Supplementary Material 1: Risk of bias assessment by category


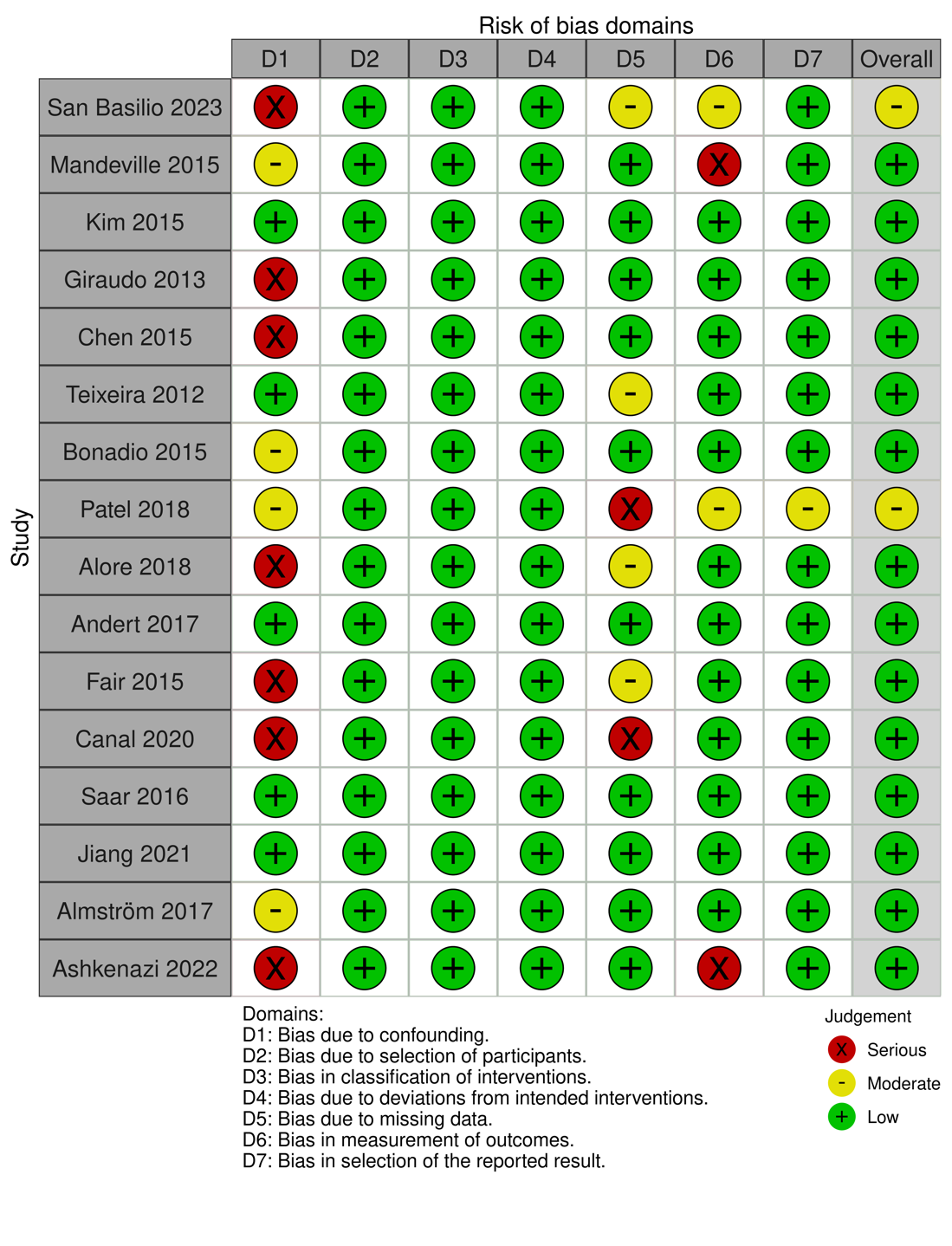


Supplementary Material 2: Risk of bias assessment by study

| 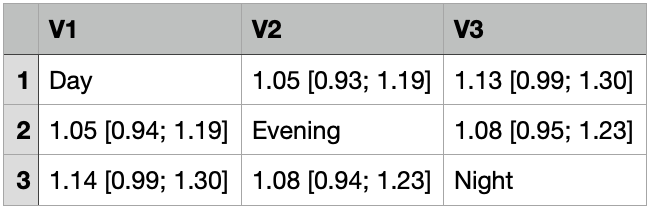 | 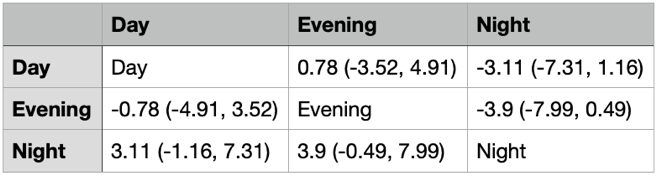 |
| --- | --- |
| 1. Post-operative complications | 1. Operative time |

Supplementary Material 3: Network-meta analysis for outcomes based on the time-of-day surgery was performed

| 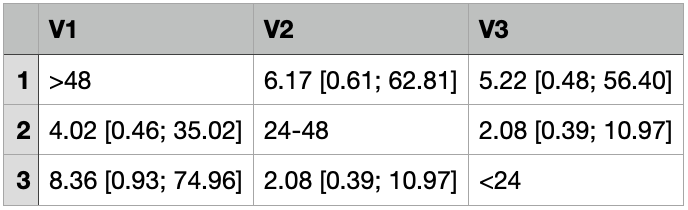 |
| --- |
| 1. Perforation rate |

Supplementary Material 4: Network-meta analysis for outcomes based on patient time

| 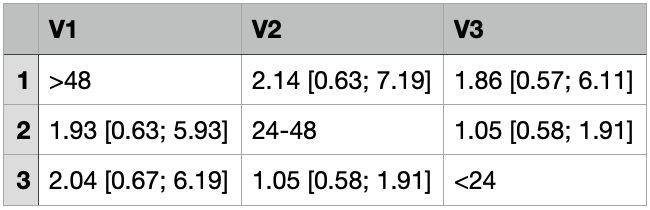 | 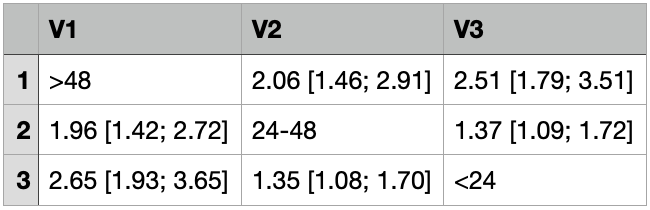 |
| --- | --- |
| 1. Perforation rate | 1. Post-operative complications |
| 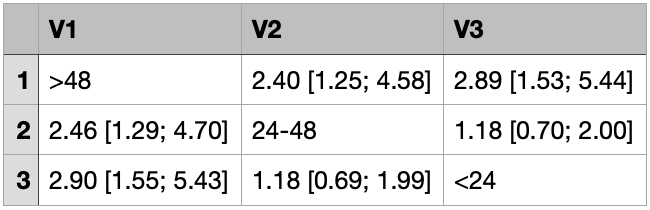 | 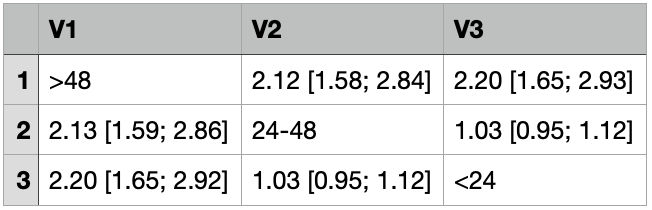 |
| 1. Surgical site infection | 1. Readmission rates |
| 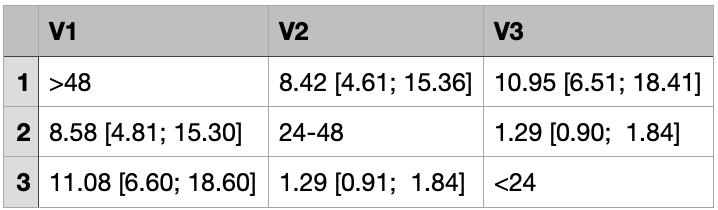 |  |
| 1. Mortality |  |

Supplementary Material 5: Network-meta analysis for outcomes based on hospital time

| 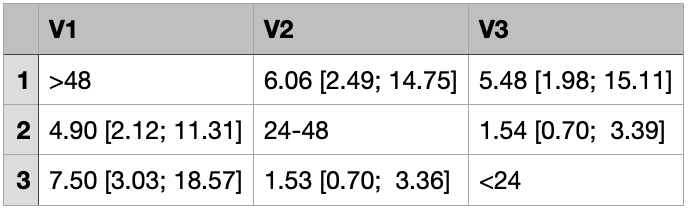 | 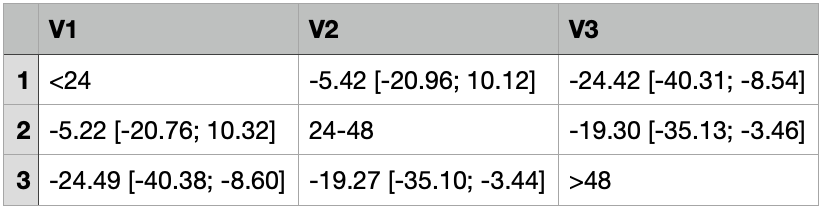 |
| --- | --- |
| 1. Perforation rate | 1. Operative time |
| 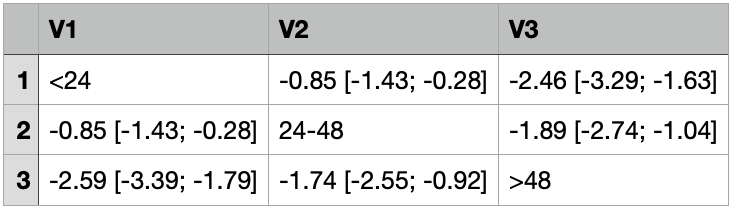 |  |
| 1. Length of stay |  |

Supplementary Material 6: Network-meta analysis for outcomes based on total time
